# A study on the stability of ritonavir form III processed in orbit and returned to Earth

**DOI:** 10.1038/s41526-026-00594-0

**Published:** 2026-04-08

**Authors:** Haley C. Bauser, Pamela A. Smith, Stephan. D. Parent, Larry R. Chan, Ami S. Bhavsar, Kenneth H. Condon, Andrew McCalip, Jordan M. Croom, Dale K. Purcell, Susan J. Bogdanowich-Knipp, Daniel T. Smith, Brett A. Cowans, Ruba Alajlouni, Stephen R. Byrn, Adrian Radocea

**Affiliations:** 1 Varda Space Industries, El Segundo, CA USA; 2 Improved Pharma LLC, West Lafayette, IN USA

**Keywords:** Chemistry, Drug discovery, Engineering, Medical research

## Abstract

Despite notable progress in realizing the benefits of microgravity, the physical stability of therapeutics processed in space has not been sufficiently investigated. Environmental factors including vibration, acceleration, radiation, and temperature, if not addressed, could impact the feasibility of in-space drug processing. The presented work demonstrates the successful recovery of the metastable Form III of ritonavir generated in orbit. The samples processed in orbit, along with control samples of Form I, Form II, Form III, and amorphous ritonavir brought to space and back exhibited excellent physical and chemical stability when exposed to space environments. Stability was determined through comparison to reference samples processed and held on Earth. By providing a detailed experimental dataset centered on survivability, we address a key concern in pharmaceutical processing under microgravity with potential applications for both drug development on Earth and pharmaceutical needs for long-duration human exploration initiatives.

## Introduction

Early proof-of-concept demonstrations conducted on parabolic flights and on extended microgravity platforms such as the International Space Station (ISS) have demonstrated the potential benefits of in-space microgravity crystallization for better understanding polymorphism and for supporting pathfinding routes towards novel formulations^1–5^. Microgravity has been demonstrated to improve the quality and particle size distribution of biologics and impact the polymorphic outcome in small molecules^1,4,5^. Polymorphic control of active pharmaceutical ingredients is a key concern for safety, manufacturability, and dosing^6,7^. Polymorphism is the ability of a molecule to crystallize with different lattice formations, each with a distinct set of thermophysical attributes, including different melting points, mechanical properties, heat capacities, as well as solubilities. Isolation of metastable forms is a routine aspect of polymorph screening, and though most often the stable polymorph is preferred for final drug products, metastable forms can be selected to improve dosing profiles or serve as enabling intermediates in manufacturing^8,9^. Unexpected interconversion from one form to a previously undiscovered, more stable form is often an unwanted scenario, most famously exhibited in the recall of ritonavir^7^. As a result, understanding stability and form conversion risks in space environments is a key concern for future space-based medicines and microgravity development^10^.

Stability testing of pharmaceuticals stored on the International Space Station has highlighted that over a period of two years, a shift in potency was observed – though well within the expected shelf-life of the pharmaceuticals^10–15^. Prior studies on small molecules were limited in scope and focused primarily on chemical degradation during extended storage in orbit or specific polymorphic outcome without in-depth stability analysis^5^. The work raised additional questions to be addressed with solid-state characterization of drug materials through standard means employed for pharmaceuticals, namely DSC, Raman and XRPD to understand the solid form evolution. Furthermore, questions on the influence of launch and reentry on the state of drug materials were not sufficiently addressed. Previous studies have been hampered, at least partially, by the limited and infrequent retrieval of materials from orbit and so extensive post-flight characterization to assess both the polymorphic state and chemical stability of pharmaceuticals processed has not yet been shown^16–18^. The presented work is the first of its kind to analyze the stability of a pure active pharmaceutical ingredient (API) processed in space as prior stability studies focus on marketed pharmaceuticals with their respective excipients and shelf stability mechanisms fully incorporated. By analyzing the stability of the pure API, we can examine the feasibility of in-orbit pharmaceutical development.

Because solubility is a critical parameter that impacts dosing, unexpected changes in polymorphic outcome can lead to safety challenges. The transformation from one crystal structure to another is governed by both thermodynamics, and kinetics. While over time a drug substance will evolve towards its lowest energy crystal structure, the rate of change can be governed by activation barriers due to the energy required for molecules to translate, rotate, and reconfigure into a different crystal structure. In general, the larger the energy difference between a metastable form and the most stable form, the stronger the driving force for conversion.

For the present work, the HIV protease inhibitor ritonavir was selected for its particularly challenging polymorphic landscape which enables assessment of form interconversion for small molecules crystallized in space^7,19,20^. An additional factor for its selection is its suitability for melt/cool crystallization^18,19^. The crystallization process was tuned for the production of the metastable Form III from the melt of stable Form II. Form III is the crystalline form most vulnerable to form conversion as compared to the other known anhydrous polymorphs of ritonavir. Form III has lower stability, but higher solubility than Form I and Form II^19^. Form III is an excellent case study for microgravity crystallization and helps address the question of whether metastable forms made in microgravity can be safely recovered after reentry. Process development details were previously published^19^. Extended stability studies on the impact of storage, humidity, and force on the metastable Form III are given in the Supplementary Materials (SM). The crystallization process was developed to produce the metastable Form III from the melt of stable Form II. Because Form III is the highest energy crystalline form of ritonavir, it’s also the form most vulnerable to form conversion.

## Results

### Overview of experiment

**Fig. 1:**
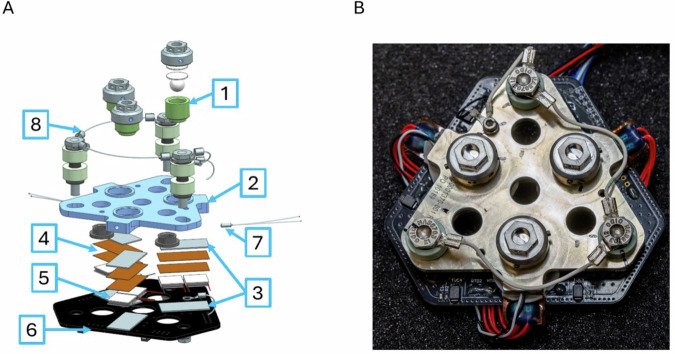
Overview of the in-orbit crystallization hardware. **A** Exploded view of the Varda PCB Stack and API vials. The components are as follows. (1) (3x) 316 Stainless Steel vials each holding ~150 mg of API, sealed with a PTFE ball and spacers. (2) 6061 Aluminum plate heat spreader holding the vials and the resistance temperature detectors (RTDs). (3) Thermal interface material (4) (6x) Kapton film heaters each with a total power of 23 watts (5) (6x) Peltier devices used in both forward bias for cooling and reverse bias for additional heater power (6) PCB (7) (2x) RTDs in heat spreader and 1x RTD on the ballast side of the PCB 8) grounding wires. **B** Picture of the fully assembled Varda PCB Stack.

The presented work demonstrates the successful recovery of the metastable Form III of ritonavir generated in orbit. The samples processed in orbit, along with control samples of Form I, Form II, Form III, and amorphous ritonavir brought to space and back exhibited excellent physical and chemical stability when exposed to space environments. Stability was determined through comparison to reference samples processed and held on Earth. Figure 1 shows an exploded rendering and photo of the crystallization hardware contained within the spacecraft. The hardware features three 0.15 mL stainless steel sample vials sealed with a stainless steel screw-on cap. A polytetrafluoroethylene (PTFE) sphere is seated between the cap and the ritonavir Form II powder to enable sealing of the powder under compression. The hardware is controlled with a printed circuit board (PCB) that enables the application of pre-programmed thermal profiles, established from ground-based studies on ritonavir’s metastable Form III^19^. Temperature control is achieved through the use of film heaters and a Peltier device in thermal contact with the flight vials via an aluminum heat spreader.

Sample loading took place on October 21^st^, 2022. Flight vials were loaded with ~150 mg of ritonavir Form II. In addition to the three vials that underwent crystallization, four ritonavir control samples were placed in the capsule, thermally isolated from the crystallization hardware to ensure that no thermal profile would be applied to the controls. The purpose of these vials was to determine if any environmental factors experienced throughout the capsule’s lifetime influence the final form of ritonavir. Examples of factors that could induce form conversion are vibration and shock events on ascent, radiation from the orbital environment, shock events during reentry, and unwanted temperature increases throughout the process of re-entering the atmosphere and returning to Earth^21,22^. Control Vial 1 contains amorphous ritonavir, Control Vial 2 contains Form I ritonavir, Control Vial 3 contains Form II ritonavir, and Control Vial 4 contains Form III ritonavir. The order of ritonavir’s form stability is amorphous < Form III < Form I < Form II^19,20^.

**Fig. 2:**
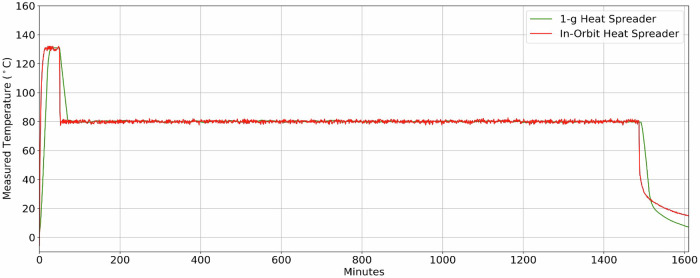
Temperature profile in 1-g and in-orbit. Comparison of the heat spreader temperature during the 1-g crystallization of ritonavir and the heat spreader temperature during the in-orbit crystallization of ritonavir.

In-orbit crystallization is performed inside a compact, unmanned capsule with Earth reentry capabilities. The reentry capsule and on-board crystallization hardware were developed by Varda Space Industries. Power, communication, and propulsion are provided by a Pioneer satellite bus (Rocket Lab, Inc.). The spacecraft was brought to orbit on a SpaceX Falcon 9 rocket on Transporter 8 launched June 12^th^, 2023. In-orbit crystallization experiments initiated on June 29^th^, 2023 across a perigee altitude of 515 km and an apogee altitude of 531 km. The melt temperature was held for 36 minutes at 131 °C + /- 2 °C. The quench from the melt temperature to the growth temperature occurred at a rate of −50.9 °C/min where it reached a temperature of 77.3 °C before stabilizing. The growth phase temperature was set at 80 °C + /- 4.2 °C for 23.97 h before cooling to 15 °C at a rate of −3.8 °C/min. Figure 2 shows the thermal profile of the terrestrial test compared to the thermal profile applied in microgravity. While remaining in orbit, the ritonavir temperature ranged from −7.5 °C to 14.5 °C.

**Fig. 3:**
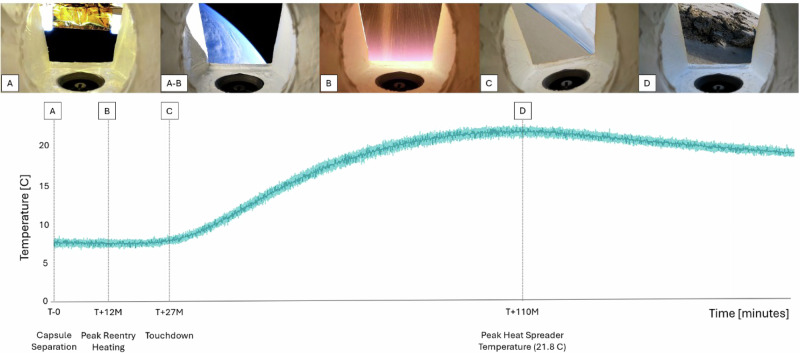
Timeline of heat spreader temperature throughout reentry with corresponding images from capsule. **A** Image and corresponding heat spreader temperature upon capsule separation from the Pioneer satellite. **A**–**B** Image shows the capsule above Earth independent of the satellite. **B** Image and corresponding heat spreader temperature at the maximum capsule external temperature during reentry. **C** Image and corresponding heat spreader temperature upon capsule touchdown in Utah. **D** Image and corresponding heat spreader temperature at maximum temperature experienced while awaiting recovery.

After in-space crystallization, the capsule remained in orbit for approximately 8 months before safely landing at the Utah Test and Training Range on February 21^st^, 2024. During reentry the test vial temperature did not exceed 23 °C as shown in Fig. 3.

In summary, the study included several controls – ritonavir processed on identical hardware on Earth, laboratory controls here on Earth, as well as on-orbit controls. Each of these controls enabled ruling out variables related to the flight hardware, A duplicate hardware set was operated back on Earth and used to confirm successful crystallization of Form III in a thermal vacuum chamber at a pressure of 0.001 Torr. Figure 2 shows the thermal profile of the terrestrial test compared to the thermal profile applied in microgravity and Table 1 summarizes the results.

**Table 1:** Summary of flight sample crystallinity

Sample ID	Starting Material	In-Orbit Crystallization	Post Reentry Material	Impurities within 0.5% of Laboratory Control
Flight Vial 1	Ritonavir Form II	Yes	Ritonavir Form III	Yes
Flight Vial 2	Ritonavir Form II	Yes	Ritonavir Form III	Yes
Flight Vial 3	Ritonavir Form II	Yes	Ritonavir Form III	Yes
Flight Control Vial 1	Amorphous Ritonavir	No	Amorphous Ritonavir	Yes
Flight Control Vial 2	Ritonavir Form I	No	Ritonavir Form I	Yes
Flight Control Vial 3	Ritonavir Form II	No	Ritonavir Form II	Yes
Flight Control Vial 4	Ritonavir Form III	No	Ritonavir Form III	Yes
Earth Control – Copy of Flight Hardware	Ritonavir Form II	No	Ritonavir Form III	No
Earth Control – Lab Hardware Ambient Storage	Ritonavir Form II	No	Ritonavir Form III	No
Earth Control – Lab Hardware Refrigerated Storage	Ritonavir Form II	No	Ritonavir Form III	N/A

### Ritonavir analysis

**Fig. 4:**
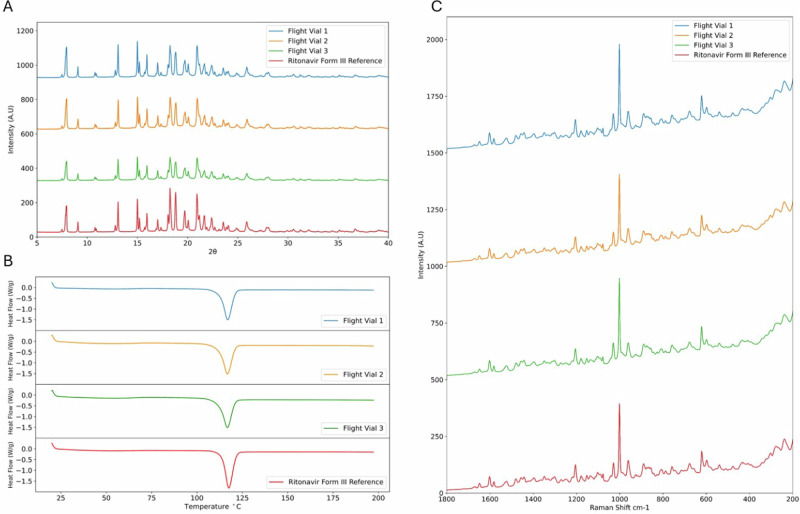
Analysis of samples crystallized in microgravity. **A** Diffractograms of material extracted from each of the three vials that underwent crystallization in-orbit. All three vials match the Form III reference material. **B** Thermograms of material extracted from each of the three vials that underwent crystallization in-orbit. All three vials match the endotherm of Form III. The melt temperatures are 116.94 °C, 116.77 °C, 116.73 °C, respectively and the heat capacities are 50.54 J/g, 51.78 J/g, and 51.11 J/g, respectively. **C** Raman spectra of material extracted from each of the three vials that underwent crystallization in orbit. The presented spectra are averages of the spectra of 10 samples from each of the vials to ensure adequate representation across the crystalline material.

Upon removal from the capsule in Utah, the crystallized and control samples were sent in a temperature-controlled environment to the Improved Pharma facility in West Lafayette, IN to analyze the material in each vial. The samples remained under refrigeration for about 2.5 days between capsule removal and commencement of analysis. Figure 4 shows the X-ray powder diffraction (XRPD) data and Raman spectra of each of the flight vials compared to a reference of Form III. The analysis indicates that all samples crystallized in microgravity are consistent with crystalline Form III. Some amorphous background is detectable in the diffraction pattern of the flight vials. We determined that this amorphous background is likely introduced in the process of sample retrieval from the vials and sample preparation for XRPD as opposed to being introduced via factors from orbit or reentry. We were able to do so by isolating crystalline material above the PTFE ball which was removable while remaining intact. XRPD data of this confirmation is shown in the SI. Furthermore, evidence of amorphous material is not detected in the differential scanning calorimetry (DSC) thermogram indicating that the samples are predominantly crystalline Form III.

**Fig. 5:**
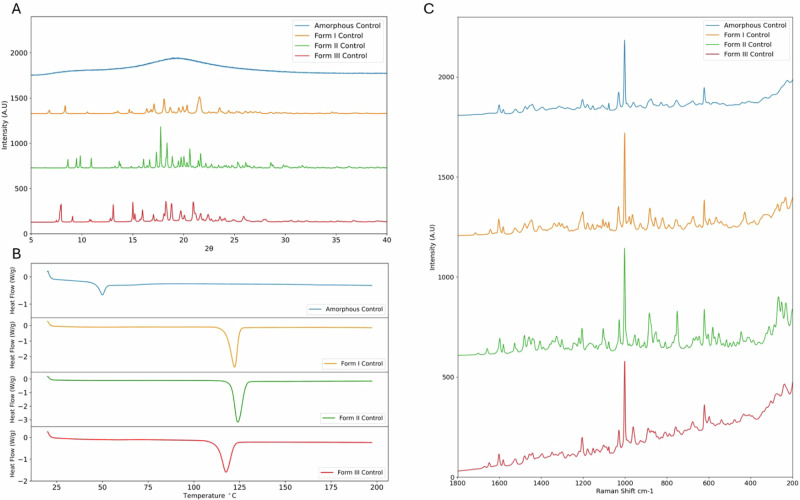
Analysis of in-orbit control samples. **A** Diffractograms of material extracted from the control vials. All diffraction patterns match that of their initially packed form. **B** Thermograms of material extracted from each of the control vials. All endotherms match that of their form as packed. The melting temperatures are 50.0 °C, 122.25 °C, 124.00 °C, and 117.52 °C, respectively. The heat capacity of Form I is 76.46 J/g, the heat capacity of Form II is 94.23 J/g, and the heat capacity of Form III is 53.13 J/g. **C** Raman spectra of material extracted from the control vials. All spectra match that of their initially packed form. The presented spectra are each an average of the spectra of 10 samples from each of the vials to ensure adequate representation across the crystalline material.

Figure 5 shows the XRPD diffractograms, Raman spectra, and DSC thermograms of the control vials that did not undergo crystallization in microgravity. All control samples, regardless of relative physical stability, were found to be of the same crystal form as packed without detectable amorphous background. This indicates that neither in-orbit radiation nor conditions upon reentry cause polymorph conversion of ritonavir or crystallization of the amorphous ritonavir.

To analyze chemical purity and degradation of the samples, we performed high-performance liquid chromatography (HPLC). The total impurities of the starting material, USP lot M-RIT/0804007 Form II ritonavir, were determined to be at 0.6%. Impurities in Form I were found to be at 0.5% when stored under refrigeration at 5 °C and 5.3% when stored in ambient. The process of melting the ritonavir to generate Form III according to the previously reported procedure introduces additional impurities to the material^19^. The impurity levels of ritonavir Form III grown terrestrially following a standard lab procedure and stored under refrigeration at 5 °C were determined to be 1.5%. When stored in an ambient environment, the total impurities reached 4.0%. As an additional point of reference, we performed HPLC measurement on two samples of Form III grown terrestrially in flight-like hardware, and their impurity levels were at 2.0% and 2.1%, respectively. Since amorphous ritonavir is also prepared via melt, there is a similar process-based introduction of impurities and therefore the measured impurities were at 1.4% when stored under refrigeration at 5 °C and 1.8% when stored in ambient.

The total impurities for flight vials 1, 2, and 3 were 1.7%, 1.7%, and 1.5%, respectively. Furthermore, the retention times of the observed impurities in the HPLC chromatograms were the same across both flight vials and laboratory controls, indicating no unique impurities were generated in space. The control vials were similarly analyzed via HPLC to detect if impurities were introduced throughout orbit and/or reentry. The impurity levels in the amorphous control vial were measured to be at 1.0%. The impurities in the Form I control vial were measured to be 0.6%. The impurities in the Form II control vial were measured to be 0.6%. The impurities in the Form III control vial were measured to be 2.0%. Similarly, no impurities outside those observed in the laboratory grown samples were detected in the in-orbit control vials. The HPLC analysis indicates that throughout orbit and reentry, no additional impurities were introduced to the ritonavir samples in any form. Table 1 summarizes the result of all vials in the W-1 capsule compared to their terrestrially grown controls.

## Discussion

Our presented results demonstrate the feasibility of processing pharmaceuticals in microgravity, building a pathway towards in-orbit manufacturing of pharmaceuticals. Our experiments address stability, which is a critical step towards derisking in-space drug development and manufacturing. While studies have shown form evolution during reentry, in contrast, we show how robust hardware design and thermal control unlock in-space production and return of even metastable forms from space^5^ Autonomous operation and reentry expands access to in-orbit processing of pharmaceuticals, as a higher cadence than that which is currently accessible via the ISS is required for applicability to the current pharmaceutical development landscape. The presented work demonstrates excellent thermal control both in-orbit and throughout recovery. For the thermal profile investigated, the polymorphic outcome for ritonavir crystallized from its melt is unchanged when compared to results on Earth. This result is due to the underlying crystallization mechanisms as well as the selection of a process that strongly favors formation of Form III^19,23,24^. Future work will examine polymorphic outcomes in microgravity by not only examining additional molecules, but also by expanding the range of thermal profiles examined, including probing behavior at the interface between known or anticipated polymorphic outcomes. The results highlight the importance of careful considerations of crystallization kinetics, thermophysical properties of crystals and their melts, including density, viscosity, and diffusion coefficients alongside ground-based studies to help inform process sensitivity to gravitational forces^25–27^. Furthermore, future work would be aided by additional terrestrial radiation tests to evaluate the limits of ritonavir’s stability against different radiation sources across a span of exposure durations with varying degrees of shielding. By demonstrating physical and chemical stability, this work enables a path towards in-space processing of pharmaceuticals that not only enables the development of novel drug products for use on Earth, but also contributes to the feasibility of long-duration human exploration initiatives during which pharmaceutical stability is crucial for safety and mission success.

## Methods

### Preparation of ritonavir

Form II ritonavir was sourced from USP lot M-RIT/0804007. In the preparation of Form I, 206.3 mg of Form II ritonavir was dissolved in approximately 6 mL or more of ethyl acetate (EtOAc) with the application of heat, approximately 70 °C. The solution was then allowed to cool to room temperature and left to stand overnight without any observable change. Subsequently, a rapid evaporation process was employed to reduce the volume by approximately half. To this concentrated solution, cold hexane (approximately 14 mL) was added slowly with continuous stirring, resulting in the immediate formation of a white precipitate which then transitioned into a sticky mass. The mixture was then vigorously vortexed, slightly warmed, and stirring was maintained at room temperature. Afterward, the mixture was placed in the freezer overnight. The resulting precipitant was recovered with filtration through a Swinnex filter assembly equipped with a nylon membrane of 25 mm diameter and 0.2 µm pore size.

In the preparation of Form III, 651.4 mg of Form II ritonavir was evenly spread on a glass microscope slide and compressed to an approximate thickness of 1 mm. The slide was then placed in an oven set at roughly 130 °C, where the solid melted completely within about 25 min. After melting, the slide was transferred to an oven maintained at 80 °C and left there for approximately 24 h. Upon removal, the sample was cooled to room temperature, and the presence of some crystals within the glass matrix was confirmed under a microscope. The sample was then returned to the 80 °C oven overnight, but no further changes were observed microscopically. Subsequently, the sample was placed back in the 130 °C oven, where it melted in roughly 1 min. The oven temperature was then gradually reduced to approximately 83 °C, and the sample was left overnight. The following day, the white to light tan solids were removed from the oven and cooled to room temperature. The sample appeared fully crystalline under microscopic examination. The final step involved removing the crystalline material from the slide and gently crushing it into a fine powder.

In the preparation of the amorphous ritonavir, 904.8 mg of Form II ritonavir was placed on a glass microscope slide and compressed to approximately 1 mm thickness. The slide was then placed in an oven preheated to approximately 130 °C, where the solid melted completely within approximately 10 min. Following this, the molten sample was rapidly quenched on a cold aluminum block taken from the freezer. The sample was then carefully removed from the slide.

Each sample, weighing 150 ± 1 mg, was carefully transferred into vials within a glove bag purged with inert, dry nitrogen gas to ensure an oxygen- and moisture-free environment.

### XRPD

XRPD patterns were collected on a PANalytical Empyrean diffractometer using a Cu Kα incident beam of radiation generated at 45 kV / 40 mA. A silicon standard was analyzed to verify the observed position of the Si <111> peak is consistent with the National Institute of Standards and Technology (NIST) certified position. Powder samples were sandwiched between 3-µm-thick Etnom films and analyzed in transmission geometry. The X-ray source was configured with Soller slits of 0.02 radians, a fixed anti-scatter slit of 1/2°, a mask of 20 mm, and a fixed divergence slit of 1/2°. The diffracted beam passed through a 3.0 mm anti-scatter extension and Large Soller slits of 0.02 radians to the detector. A beam-stop was used to minimize the background generated by air. Diffraction patterns were collected with Data Collect or software using a PIXcel3D-Medipix3 detector located 240 mm from the specimen. The data was acquired using a single scan from 2–50° 2θ with the sample spinning at a revolution time of 2 s.

### Raman spectroscopy

A HORIBA Scientific XploRA Series Confocal Raman Microscope (Piscataway, NJ) was used to collect Raman spectra using the following parameters: 785 nm laser at 100% power, 1200 g/mm grating, 300 micrometer confocal hole, 100 micrometer slit entrance to the spectrograph, 1 second spectra acquisition with 30 accumulations. The Raman signal is detected using a Syncerity Model 356399, thermoelectrically cooled CCD detector. Spectra were acquired over the range −125 to 1800 cm^−1^. An Olympus Series BX51TRF polarized light microscope (Olympus America Inc., Melville, NY) provided the base optical platform. An Olympus MPlan N Series 20X, 0.40 NA microscope objective was used to focus the laser light onto the sample and to collect the Raman signal. The microscope was equipped with a Marzhauser Wetzlar computer-controlled mapping stage to translate the sample for focus and data acquisition. Digital images were acquired using a Lumenera Series Infinity 3-1 C (Teledyne Lumenera, Ottawa, Ontario, Canada) camera using Infinity software version 6.5.6 and Infinity Analyze software version 7.0.2.930 (Build data 1-May-2020). System calibration was performed prior to analysis using a silicon disc to monitor peak position at 520.7 cm^−1^.

The sample was prepared for analysis by placing a small amount of material onto a gold-coated microscope slide using a tungsten needle and dispersed to a thin layer. The small sample was illuminated with white light using 200x magnification for specific sample area analysis.

### DSC

DSC was performed using a TA Instruments model Q10 differential scanning calorimeter. The instrument was calibrated using indium. The sample was placed into a standard aluminum DSC pan, covered with a lid that was manually pierced with a pin, and the weight was accurately recorded. The pan lid was crimped prior to sample analysis. An aluminum pan configured as the sample pan was placed on the reference side of the cell. The sample was analyzed in a single run from 20 to 200 °C at a heating rate of 10 °C/min under a purge of nitrogen (50 cc/min).

### HPLC

The HPLC method was adapted from Yekkala et al.^28^.

### Mobile phase preparation

Phosphate buffer was prepared by adding 6.9 g of sodium phosphate monobasic and 0.8 g of heptanesulfonic acid to ~800 mL purified water and adjusting to pH 4.0 with ortho-phosphoric acid. This solution was brought to volume (1 L) and subsequently used to prepare both mobile phases which consisted of Mobile phase A: ACN:buffer:water (35:28:37) and Mobile phase B: ACN:buffer:water (70:28:2). The resulting mobile phases were degassed in an ultrasonicator.

### Diluent preparation

Diluent was prepared by combining mobile phase A and mobile phase B (70:30).

### Standard preparation

A 0.5 mg/mL stock ritonavir reference standard solution was made by accurately weighing 5.4 mg into a 10 mL volumetric flask and diluting to volume with diluent.

### Sample preparation

Each sample to be analyzed was made by accurately weighing approximately 5 mg into a 10 mL volumetric flask, diluting to volume with diluent, and mixing well. These solutions were added to an amber HPLC vial, and analyzed according to the HPLC conditions below. Each sample vial was analyzed in duplicate.

### HPLC analysis

All HPLC analyses were conducted using a gradient on an Agilent 1100 HPLC system equipped with a degasser, autosampler, quaternary pump and a UV diode array detector. OpenLab, ChemStation Edition, Rev. C01.10 [287] software was used for all analyses. Reference Yekkala et al. was used as a guide according to the following instrumental parameters^28^:

**Table:** 

Column:	Zorbax C18, 4.6 mm×250 cm, 5 µm, 80 Å
Mobile phase A:	ACN, buffer, water, 35:28:37
Mobile phase B:	ACN, buffer, water, 70:28:2
Injection volume:	25 µL
Flow rate:	1 mL/min
Column temperature:	35 °C
Detector:	240 nm (spectrum collected from 190-400 nm)
Run time:	60 min

**Table:** 

Time (min)	Mobile Phase A (%)	Mobile Phase B (%)
0	70	30
25	70	30
35	0	100
45	0	100
50	70	30

10 min post run equilibration time

## Supplementary information

Supplementary Materials

## Data Availability

The data that support the findings of this study are included in this published article and its supplementary materials.
