# Supplementary material for: A study on the stability of ritonavir form III processed in orbit and returned to Earth
Source: NPJ Microgravity. 2026 Apr 8;12:52. doi: 10.1038/s41526-026-00594-0 (PMC13250138; doi:10.1038/s41526-026-00594-0)
Supplement: Supplementary file 1 — Supplementary Materials [file 41526_2026_594_MOESM1_ESM.pdf]

Supplementary Materials for

A Study on the Stability of Ritonavir Form III Processed in Orbit and Returned to Earth

Haley C. Bauser, Pamela A. Smith, Stephan. D. Parent, Larry R. Chan, Ami S. Bhavsar, Kenneth H. Condon, Andrew McCalip, Jordan M. Croom, Dale K. Purcell, Susan J. Bogdanowich-Knipp, Daniel T. Smith, Brett A. Cowans, Ruba Alajlouni, Stephen R. Byrn, and Adrian Radocea*

Adrian Radocea

Email: adrian@varda.com

This PDF file includes:

Fig. S1
Fig. S2
Fig. S3
Fig. S4
Fig. S5
Fig. S6
Fig. S7
Fig. S8
Fig. S9
Fig. S10
Table S1
Table S2

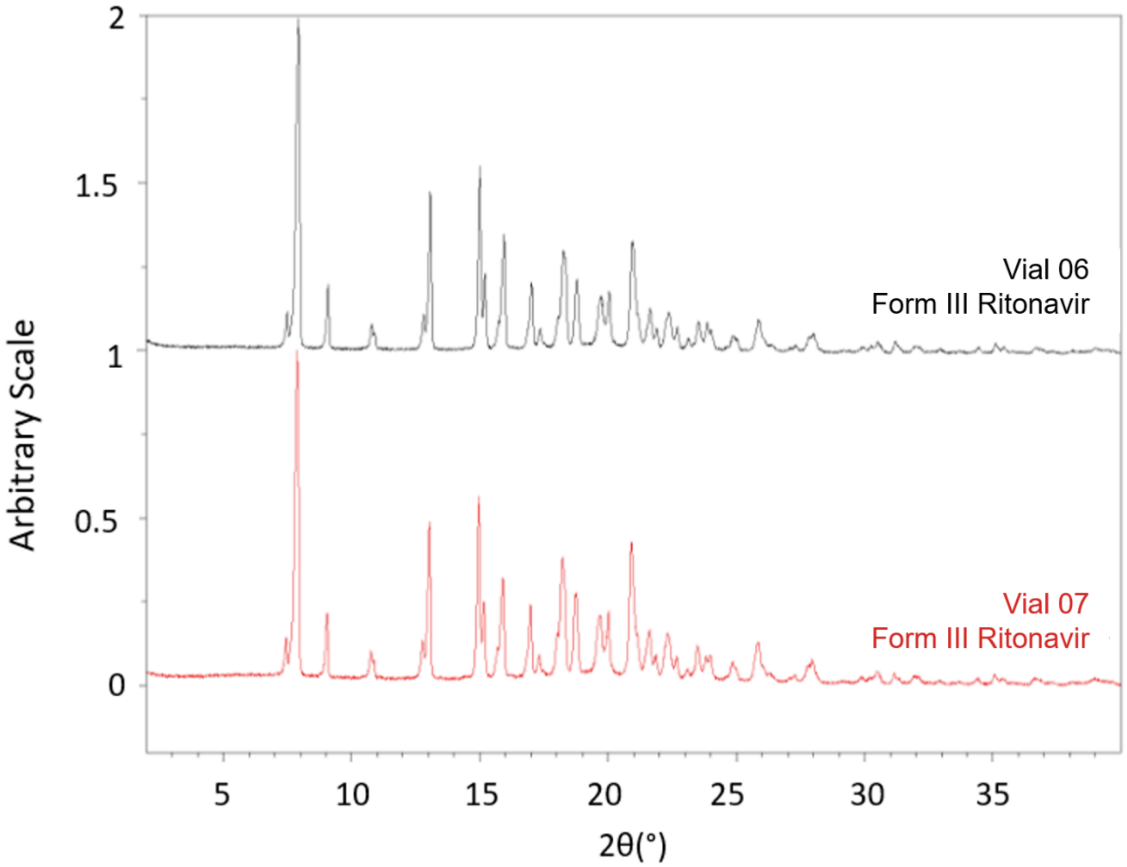

Fig. S1. Resulting Crystallinity from Terrestrial Run for Record. XRPD diffractograms of ritonavir crystallized in the payload hardware terrestrially under vacuum. Both sample patterns match that of Form III.

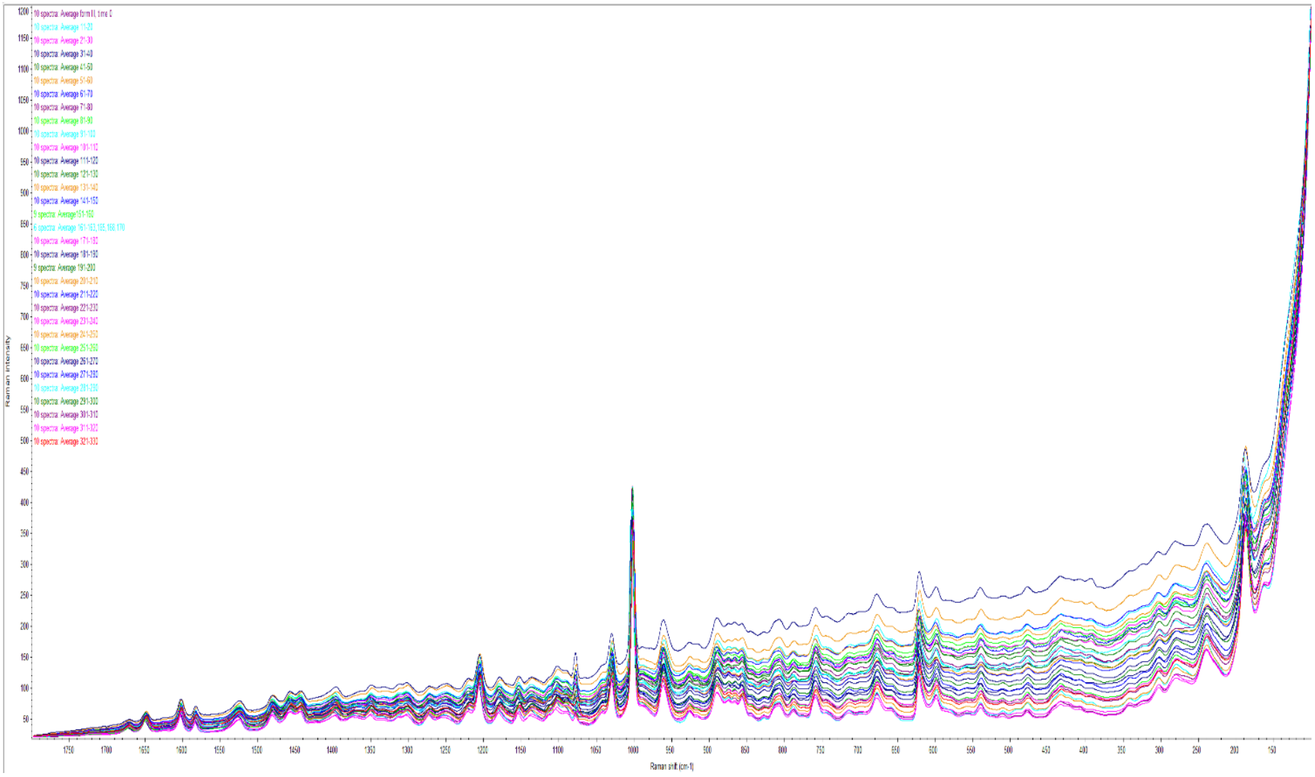

Fig. S2. Overlay of Raman spectra on Form III acquired monthly across 32 months in ambient storage since the lab control Form III was originally crystallized. Form III has not undergone any physical changes since its initial crystallization.

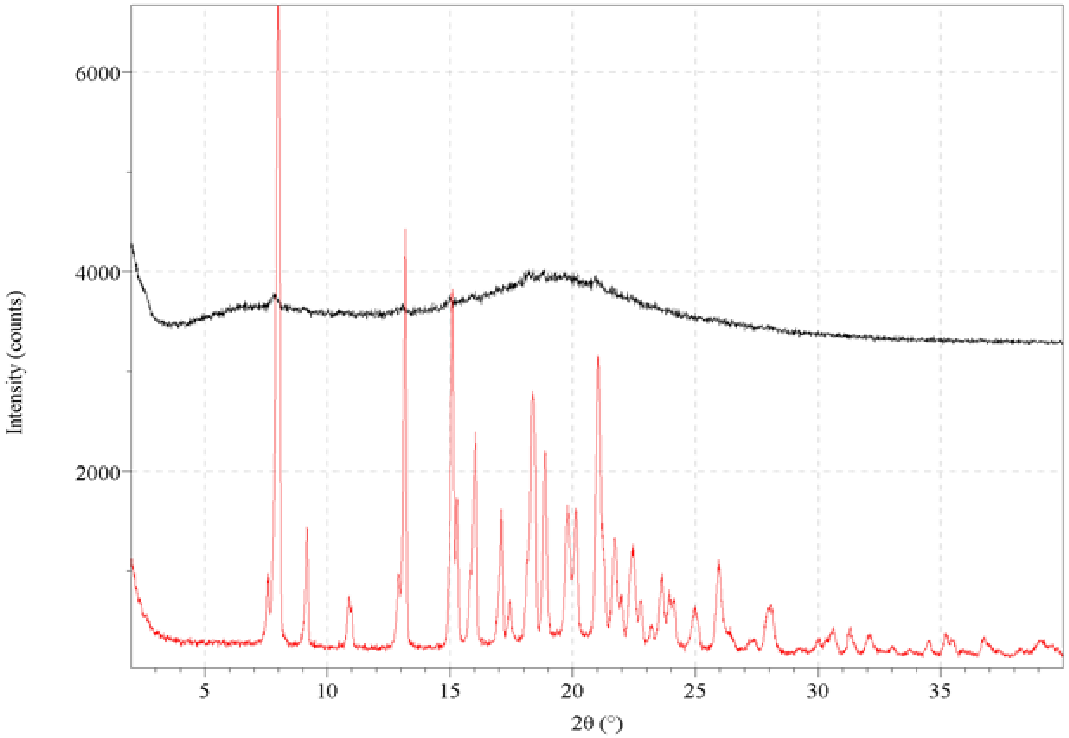

Fig S3. Form III ritonavir before and after 10 minutes of grinding with a mortar and pestle. The powder becomes predominantly amorphous**.**

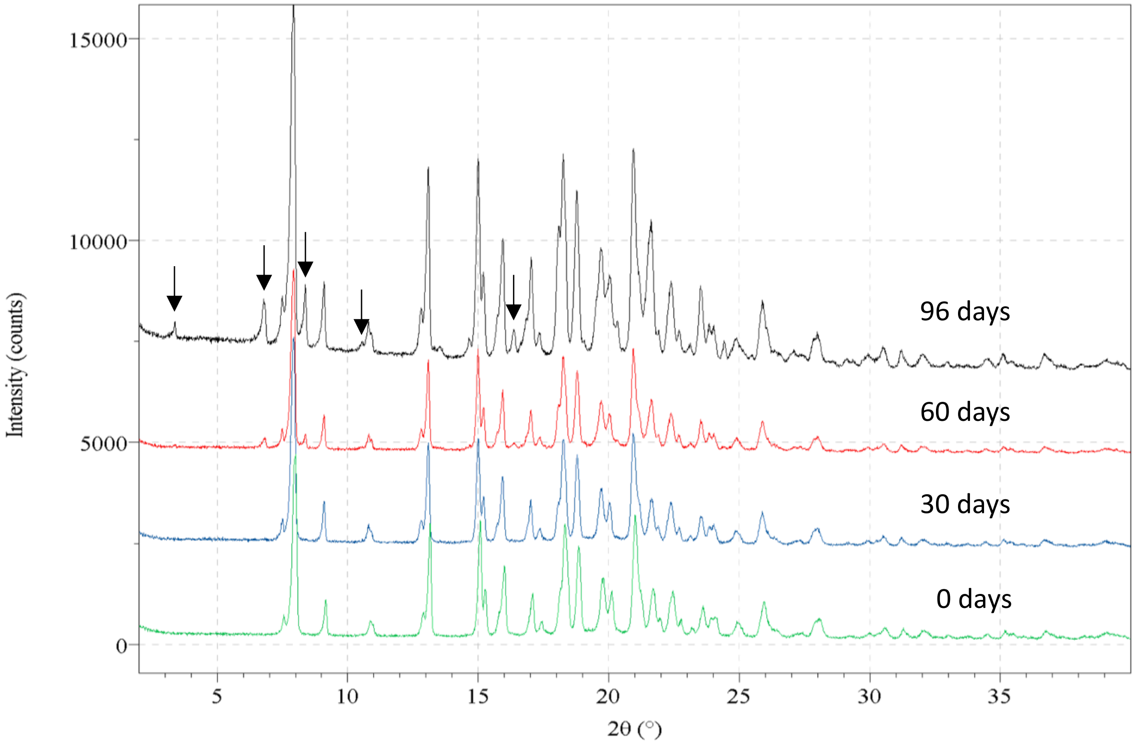

Fig S4. Form III ritonavir held in storage at 40 °C and 75% room humidity. Form I appears at the 60-day measurement and continues to grow.

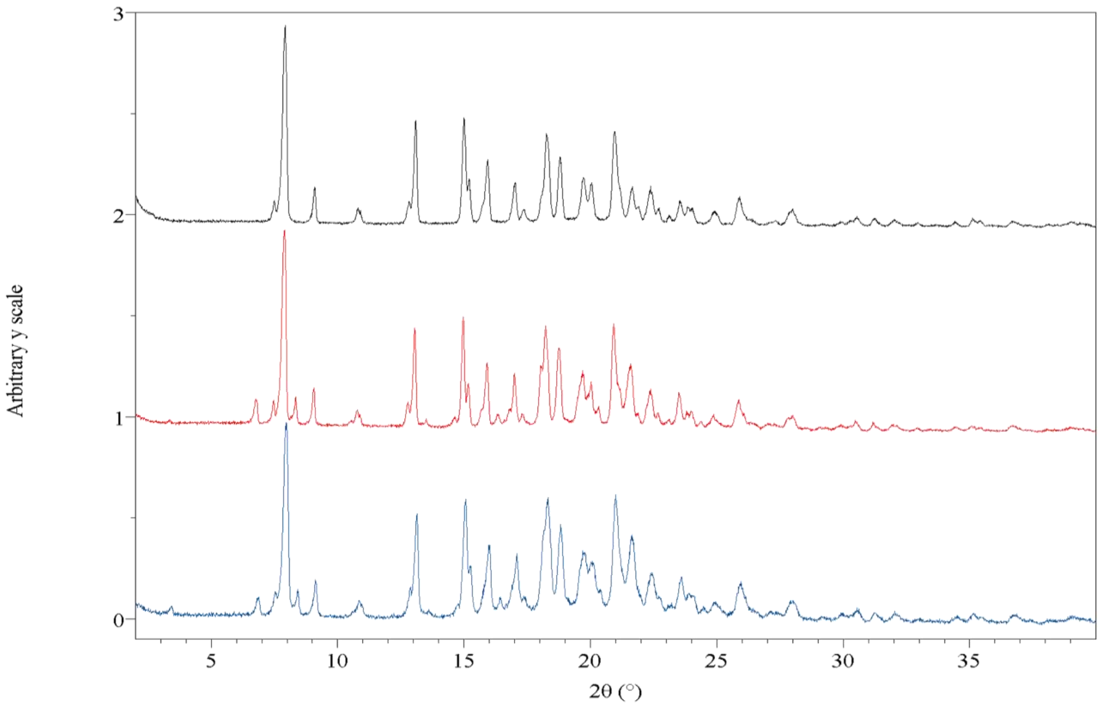

Fig S5. Three samples of ritonavir Form III that were shaken at +3dB for 15 minutes to simulate launch conditions. This experiment was performed after crystallization in flight-like vials. Form I peaks appear in the diffractogram.

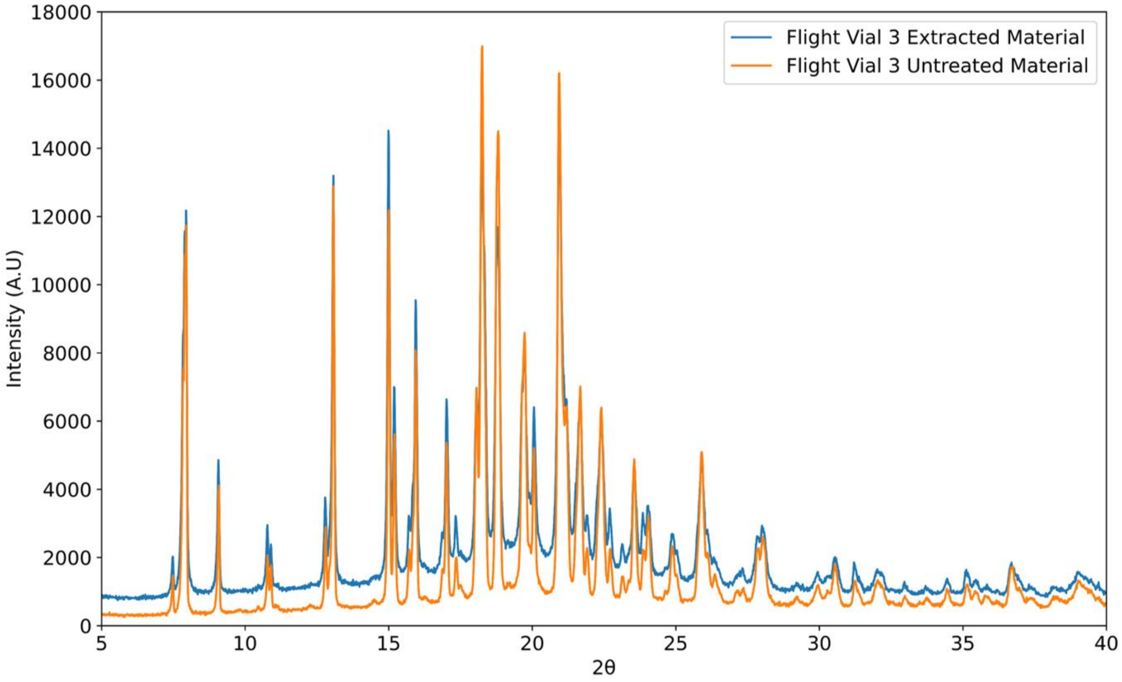

Fig. S6. XRPD of untreated material vs. extracted and lightly ground material. The diffraction pattern of the extracted material from Flight Vial 03 contains some amorphous background that can be attributed to the process of removing the materials from the vials and lightly grinding for XRPD measurement. The untreated material was removed in a large piece from the Teflon ball seal and was therefore not subjected to the same removal force as the bulk of the material from Flight Vial 3. The untreated material does not have the same amorphous background indicating the extraction is the cause of the amorphous background instead of the forces during re-entry.

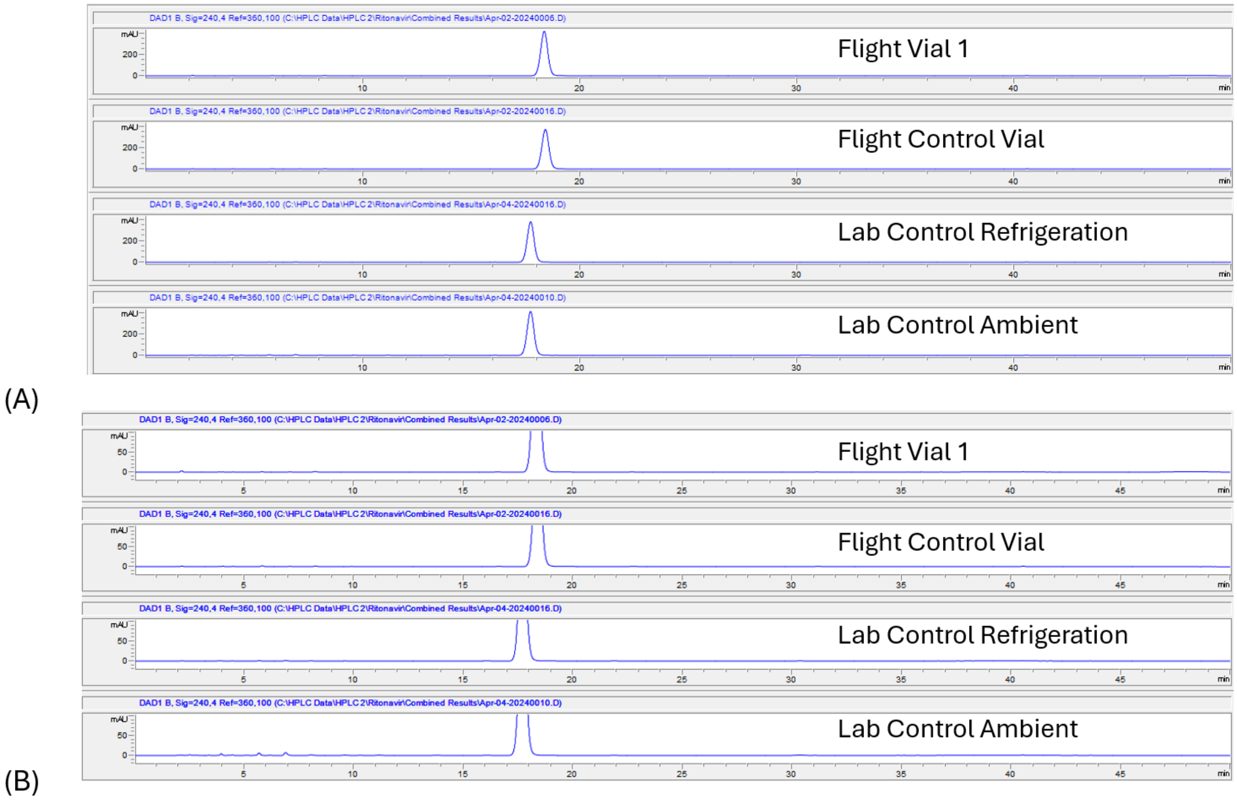

Fig. S7. Chromatograms showing the purity of samples crystallized in orbit compared to the flight and terrestrial controls. (A) shows the full chromatogram and (B) shows a zoomed in view of the impurities.

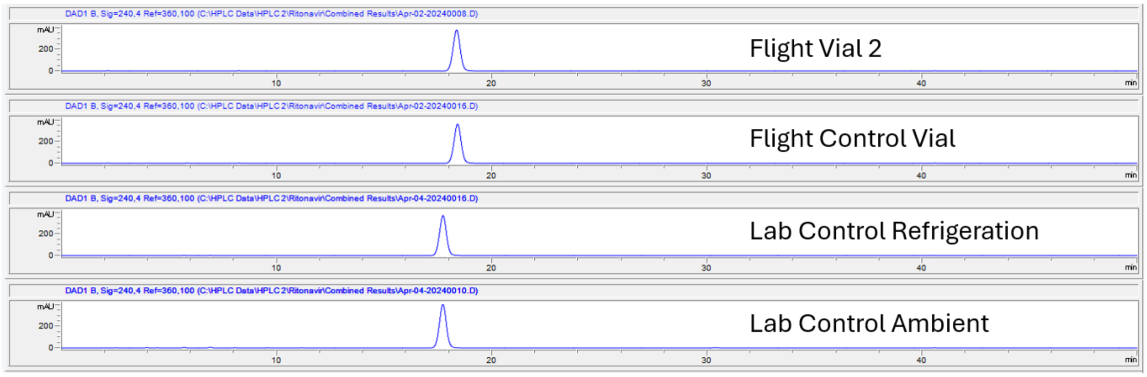

(A)

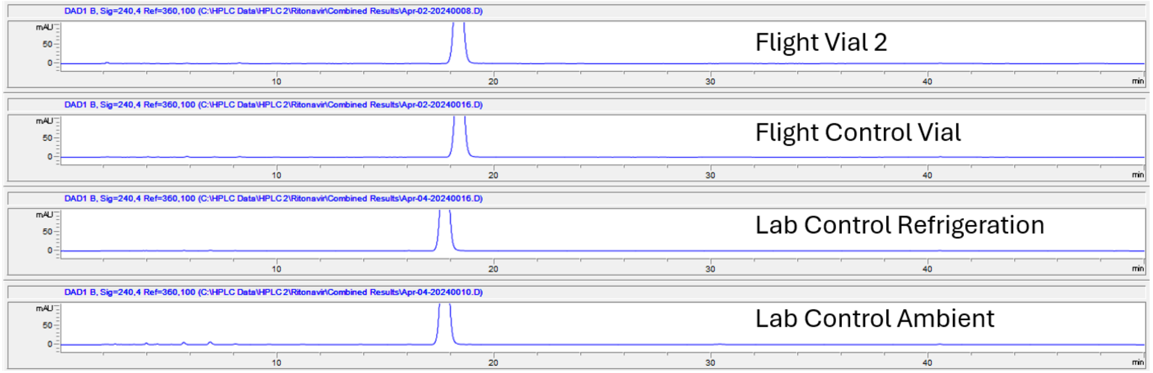

(B)

Fig. S8. Chromatograms showing the purity of samples crystallized in orbit compared to the flight and terrestrial controls. (A) shows the full chromatogram and (B) shows a zoomed in view of the impurities.

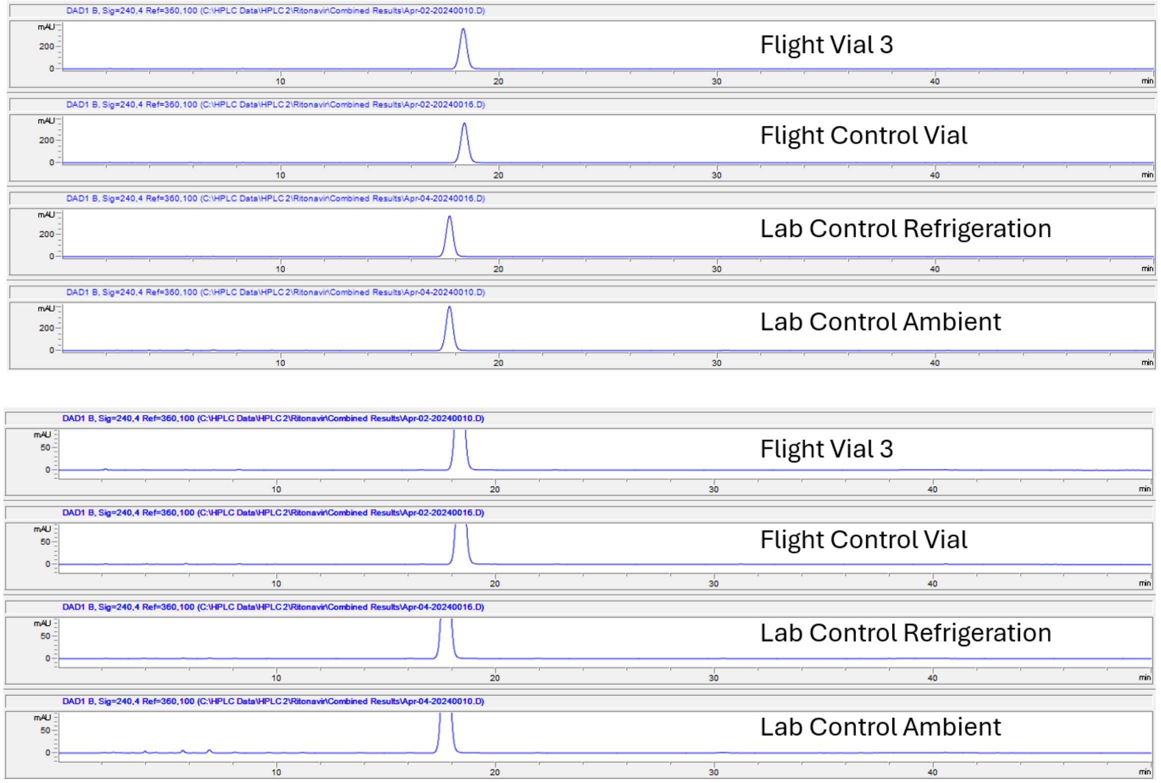

(A)

(B)

Fig. S9. Chromatograms showing the purity of samples crystallized in orbit compared to the flight and terrestrial controls. (A) shows the full chromatogram and (B) shows a zoomed in view of the impurities.

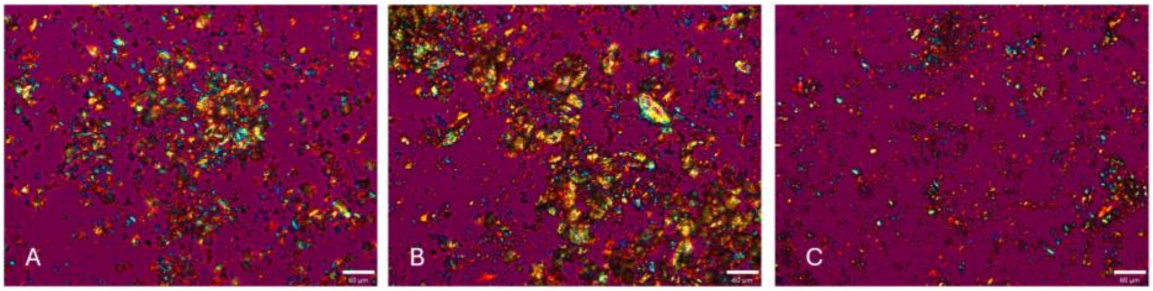

Fig. S10. PLM images of (A) Flight Vial 1, (B) Flight Vial 2, and (C) Flight Vial 3.

Table S1. Hardware requirements compared to terrestrial and in-orbit performance

		Requirement	1-g Run-for-Record	Microgravity
**Melt Phase**	**Melt Ramp Rate**	Any	11.2°C/min	9.6°C/min
	**Melt Temp**	131°C +/-3°C	130°C +/-1°C	131°C +/-2°C
	**Melt Duration**	> 20 min	38.4 min	36 min
	**Melt Quench Rate**	≤-20°C/min	-29°C/min	-50.9°C/min
	**Melt Quench Min Temp**	75°C	77.7°C	77.3°C
**Growth Phase**	**Growth Temp**	80°C +/-3°C	80°C +/-0.5°C	80°C +/-4.2°C
	**Growth Duration**	24 hours +/- 1hour	23.6 hours	23.83 hours
	**Growth Quench Rate**	Any	-4.5°C/min	-3.8°C/min

Table S2. Impurities of each ritonavir sample analyzed via HPLC and their respective duplicates

Sample	Impurity %
USP lot M-RIT/0804007 Form II, Sample 1	0.573
USP lot M-RIT/0804007 Form II, Sample 2	0.563
Form I, Laboratory Control, 5 °C Sample 1	0.543
Form I, Laboratory Control, 5 °C Sample 2	0.520
Form I, Laboratory Control, Ambient Sample 1	5.268
Form I, Laboratory Control, Ambient Sample 2	5.310
Amorphous, Laboratory Control, 5 °C Sample 1	1.434
Amorphous, Laboratory Control, 5 °C Sample 2	1.420
Amorphous, Laboratory Control, Ambient Sample 1	1.784
Amorphous, Laboratory Control, Ambient Sample 2	1.786
Form III, Laboratory Control, 5 °C Sample 1	1.445
Form III, Laboratory Control, 5 °C Sample 2	1.494
Form III, Laboratory Control, Ambient Sample 1	4.045
Form III, Laboratory Control, Ambient Sample 2	3.982
Form III, Terrestrial Run for Record, Vial 06, Sample 1	1.973
Form III, Terrestrial Run for Record, Vial 06, Sample 2	2.009
Form III, Terrestrial Run for Record, Vial 07, Sample 1	2.089
Form III, Terrestrial Run for Record, Vial 07, Sample 2	2.101
Flight Vial 1, Sample 1	1.661

Flight Vial 1, Sample 2	1.697
Flight Vial 2, Sample 1	1.670
Flight Vial 2, Sample 2	1.644
Flight Vial 3, Sample 1	1.548
Flight Vial 3, Sample 2	1.510
Amorphous Control Vial, Sample 1	1.045
Amorphous Control Vial, Sample 2	1.006
Form I Control Vial, Sample 1	0.603
Form I Control Vial, Sample 2	0.646
Form II Control Vial, Sample 1	0.585
Form II Control Vial, Sample 2	0.609
Form III Control Vial, Sample 1	2.046
Form III Control Vial, Sample 2	1.981
